# The undisciplinary journey: early-career perspectives in sustainability science

**DOI:** 10.1007/s11625-017-0445-1

**Published:** 2017-06-21

**Authors:** L. Jamila Haider, Jonas Hentati-Sundberg, Matteo Giusti, Julie Goodness, Maike Hamann, Vanessa A. Masterson, Megan Meacham, Andrew Merrie, Daniel Ospina, Caroline Schill, Hanna Sinare

**Affiliations:** 1 Stockholm Resilience Centre, Stockholm University, Stockholm, Sweden; 2 Centre for Complex Systems in Transition, Stellenbosch University, Stellenbosch, South Africa; 3 The Beijer Institute of Ecological Economics, The Royal Swedish Academy of Sciences, Stockholm, Sweden

**Keywords:** Interdisciplinary, Education, Sustainability science, Undisciplinary, Methodological groundedness, Epistemological agility

## Abstract

The establishment of interdisciplinary Master’s and PhD programs in sustainability science is opening up an exciting arena filled with opportunities for early-career scholars to address pressing sustainability challenges. However, embarking upon an interdisciplinary endeavor as an early-career scholar poses a unique set of challenges: to develop an individual scientific identity and a strong and specific methodological skill-set, while at the same time gaining the ability to understand and communicate between different epistemologies. Here, we explore the challenges and opportunities that emerge from a new kind of interdisciplinary journey, which we describe as ‘undisciplinary.’ Undisciplinary describes (1) the space or condition of early-career researchers with early interdisciplinary backgrounds, (2) the process of the journey, and (3) the orientation which aids scholars to address the complex nature of today’s sustainability challenges. The undisciplinary journey is an iterative and reflexive process of balancing methodological groundedness and epistemological agility to engage in rigorous sustainability science. The paper draws upon insights from a collective journey of broad discussion, reflection, and learning, including a survey on educational backgrounds of different generations of sustainability scholars, participatory forum theater, and a panel discussion at the Resilience 2014 conference (Montpellier, France). Based on the results from this diversity of methods, we suggest that there is now a new and distinct generation of sustainability scholars that start their careers with interdisciplinary training, as opposed to only engaging in interdisciplinary research once strong disciplinary foundations have been built. We further identify methodological groundedness and epistemological agility as guiding competencies to become capable sustainability scientists and discuss the implications of an undisciplinary journey in the current institutional context of universities and research centers. In this paper, we propose a simple framework to help early-career sustainability scholars and well-established scientists successfully navigate what can sometimes be an uncomfortable space in education and research, with the ultimate aim of producing and engaging in rigorous and impactful sustainability science.

**Electronic supplementary material:**

The online version of this article (doi:10.1007/s11625-017-0445-1) contains supplementary material, which is available to authorized users.

## Introduction

The future well-being of people and our shared Earth depend on understanding the interconnectedness of nature and society, and guiding these relationships along more sustainable pathways (Kates et al. 2001; Komiyama and Takeuchi 2006; Leach et al. 2010; Folke et al. 2016). Consequently, inter- and transdisciplinary approaches to problem-driven and solutions-oriented research have gained considerable traction over the past few decades, clearly reflected in the development of the field of sustainability science (Kates et al. 2001; Carpenter et al. 2009; Lang et al. 2012; Brandt et al. 2013; Pereira et al. 2015; Ruppert-Winkel et al. 2015).

Sustainability science is described as a field of research that brings together scholarship, policy and practice, global and local perspectives from the North and South, as well as disciplines across the natural and social sciences, humanities, engineering, and medicine (Clark and Dickson 2003). Miller et al. (2014) demonstrate that sustainability science has made progress in the past decade toward deepening our understanding of sustainability-related problems and challenges, even though large gaps remain in impact on actual sustainability transitions. While it remains unclear whether sustainability science is indeed (yet) an established scientific discipline, it is broadly recognized that a science of sustainability requires collaboration between disciplines and across theory, practice, and policy (Bettencourt and Kaur 2011). Bettencourt and Kaur (2011, p. 19540) state that ‘there is arguably no example in the history of science of a field that from its beginnings could span such distinct dimensions and achieve at once ambitious and urgent goals of transdisciplinary scientific rigor and tangible socioeconomic impact.’ To be trained as a sustainability scientist then requires new ways of engaging with each other, with the world around us, and of reflection within our own scientific processes.

Until recently, the path from disciplinary work to multidisciplinary coordination, and ultimately to inter- and transdisciplinary endeavors (definitions in Table 1) has often been explored by scholars firmly grounded in a discipline, by stepping out of their comfort zones to tackle issues that sit between disciplinary boundaries. This was the case of pioneering researchers in sustainability science and its intellectual predecessors, who often had strong roots within their disciplines, or were already established scholars, but pushed the boundaries of accepted paradigms and operated in an interdisciplinary way (e.g., as described by Folke 2006).

**Table 1:** Definitions of different types of mixed-disciplinary research

Mixed discipline research	Definition
Multidisciplinarity	Multidisciplinarity is thematically organized rather than problem-oriented. Disciplinary boundaries are generally not crossed, but rather different disciplines are considered in parallel (Stock and Burton 2011)
Interdisciplinarity	Interdisciplinarity integrates perspectives, information, data, techniques, tools, concepts, and/or theories from two or more disciplines (Cronin 2008)
Transdisciplinarity	A process of collaboration between scholars and non-scholars on a specific real-world problem (Walter et al. 2007)
Undisciplinarity	Problem-based, integrative, interactive, emergent, reflexive science, which involves strong forms of collaboration and partnership (Robinson 2008)

Consequently, sustainability science has multiple origins, which resulted in the development of various subfields over the past decades; these are characterized by different research foci, questions, vocabularies, methodologies, epistemologies, or even worldviews (de Vries 2013). For example, the ‘pathways to sustainability’ approach (Leach et al. 2010) and ‘resilience thinking’ (Folke et al. 2010, 2016) are both subfields in sustainability science with a systems perspective and an emphasis on complex human–environment interactions and notions of adaptive learning and diversity. However, their distinct origins lead to ontological and epistemological differences, which manifest themselves in different problem definitions and prescriptions of actions (West et al. 2014). With its roots in development, as well as science and technology studies, the pathways to sustainability approach tends to have human well-being and just governance as starting points from which environmental sustainability can be pursued. Resilience thinking on the other hand, with its roots in ecology (Holling 1973), is shaped by a worldview in which the biosphere is the foundation for all other interactions on Earth (Folke et al. 2016). As a group of authors, we identify ourselves more so with the latter, and by our curiosity to understand how social-ecological interconnections lead to emergent phenomena in complex systems. The framework of complex adaptive systems (Levin 1998) and concepts of resilience, adaptation, and transformation (Folke et al. 2016) are some of the theoretical underpinnings of our position in the broader field of sustainability science.

The pioneering spirit of ecological and economic scholars in the 1980s (Kates et al. 2001) has enabled an upcoming generation of sustainability scholars. Many early-career sustainability researchers focus on interdisciplinary issues from early on in their academic careers (often obtaining interdisciplinary Bachelor’s, Master’s, and PhD degrees), without necessarily developing the same strong disciplinary roots upon which previous generations of sustainability scholars built their work. This circumstance is made possible by the multitude of interdisciplinary undergraduate and graduate programs in sustainability science that have been established in recent years (see Supplementary Materials 1). We note, however, that there is still significant resistance to this profile in the academic community, both in principle—due to concerns about competency, quality, and standards of what it means to be a sustainability scholar—and in practice—due to the relatively rigid institutional structure of most universities and their employment criteria, performance metrics, and incentive systems (Robinson 2008; Whitmer et al. 2010; Brown et al. 2015; Turner et al. 2015). There is evidence and concern that students starting on interdisciplinary career tracks have more difficulty in finding jobs within academia and publish less than students graduating in traditional, disciplinary subject areas (Rhoten and Parker 2004; Leahey et al. 2012).

Given these challenges, as early-career sustainability scholars, we face a tension that arises from our common motivation to articulate, conceptualize, and address complex human–environment problems, often broad in nature, while at the same time ensuring that we develop a sufficiently specialized skill-set to contribute meaningfully toward knowledge generation, or even to articulate solutions to complex societal issues. We have summarized this dilemma in the following way:

How can we, as graduate students and future researchers, perform high-quality research and build identity in the field of sustainability science, when starting as interdisciplinary individuals without profound roots in a discipline, and working within a world dominated by established disciplines?

This dilemma recognizes the early-stage interdisciplinary training that we observe as a new feature of our academic generation. We find that, increasingly, PhD students like us are no longer ecologists, economists, or sociologists working together in an interdisciplinary team, but rather that we are interdisciplinary individuals engaging with disciplines, or even that from our interdisciplinary training we engage with others with a similar interdisciplinary background, collaborating in an effort to create inter- and transdisciplinary science, and essentially practicing what we refer to here as ‘undisciplinary science’ (Robinson 2008). Robinson (2008) outlines undisciplinary science as problem-based, integrative, interactive and emergent, reflexive and involving strong forms of collaboration and partnership. This definition is focused largely on transdisciplinary science involving participation of academic and non-academic actors (Table 1). While many sustainability scholars engage in such transdisciplinary work, in this paper we are concerned primarily with the generation of knowledge at the intersection of existing disciplines within academia. In this study, we build upon Robinson’s definition and explore the undisciplinary journey as a descriptive feature of the space or condition at the beginning of a research career with interdisciplinary training/education, as well as a suggested prescription for how to navigate the process toward developing a foundation for rigorous interdisciplinary sustainability science. Through a reflexive process, an undisciplinary orientation may be developed, guiding one’s approach to sustainability science endeavors. These three phases are what we refer to as the ‘undisciplinary journey,’ which we explore in this paper using three distinct methods: a survey on educational backgrounds of different generations of scientists, participatory forum theater, and a panel discussion at the Resilience 2014 conference (held in Montpellier, France).

Through these combined activities, we explore what it means to practice sustainability science in an undisciplinary space at an early stage of our careers, and address the following key points:

1. What does undisciplinary mean (to us/others)?
2. What challenges and opportunities come with doing ‘undisciplinary’ research?
3. How do we address these challenges and take advantage of these opportunities within our current institutional structures?

As a group of young scholars at a sustainability science institution, the authors of this paper feel well situated to reflect on the formal and informal dimensions of this process. We hope that the lessons we have learned will be of use to other early-career scholars faced with similar opportunities and challenges.

## Methodological approach

In order to explore what it means to be a generation of early-career sustainability scholars facing new challenges and the particular dilemma outlined in the introduction, we embarked upon three phases of inquiry designed to take advantage of the Resilience 2014 conference in Montpellier, France—a major international event in the field of sustainability science that is held every three years. Resilience thinking is a subfield of sustainability science (Folke et al. 2016), which acknowledges the complex interlinkages and dependencies between social, economic, and ecological systems, at multiple scales (Folke et al. 2002; Folke 2006; Xu et al. 2015), and is increasingly becoming an integral part of practice, policy, and theory (Folke et al. 2016). The conference was an ideal sampling space since the participants of this conference captured a broad spectrum of sustainability scholars within the broader resilience research and practice network/community, from different backgrounds and levels of experience. The three steps in our inquiry were: (1) a survey within the broader resilience science community; (2) participatory forum theater, first in an exploratory workshop within the PhD student cohort at our institution and then at an open conference session, and (3) an expert panel discussion. The latter two steps took place at the Resilience 2014 conference in a special session called ‘Students’ perspectives on sustainability science research—are we moving towards undisciplinarity?’

### A survey of interdisciplinarity in sustainability science

We developed an online survey to explore the (inter)disciplinary backgrounds of sustainability scientists in the broader network of resilience research and practice, and how these vary across different generations of researchers. We sent out the survey link to the e-mail list of all Resilience 2014 conference participants (over 800 in total), as well as different social media platforms (Twitter, Facebook, resilience focused blogs such as *Resilience Science*, and the Resilience Alliance newsletter), prior to the conference. We received a total of 385 replies between April 9 and 30, 2014, of which we included 325 in our analyses, as we were only interested in respondents who had already obtained a PhD degree or who were PhD candidates at the time of responding to the survey.

To be able to categorize conference participants, the survey recorded the year in which each participant obtained or expected to obtain her/his PhD degree (<1990, 1990–1999, 2000–2009, 2010–2014, >2015), as well as participants’ organizational affiliations and positions. The core of the survey included the following questions on disciplinary background and current work:

1. Which of the following options most closely resembles the title of your undergraduate degree (Bachelor or similar), as stated in the diploma?
2. Which of the following options most closely resembles the title of your Master’s degree (MSc/MA or similar), as stated in the diploma?
3. Which of the following options most closely resembles the title of your PhD degree, as (will be) stated in the diploma?
4. Which of the following options best describes your current research area?

For each of these questions, the respondents were asked to choose one option from a list of 27 disciplines (classified in the categories of Natural Sciences, Social Sciences, Humanities, Mathematics/Statistics, and Applied Sciences/Professions—following a conventional faculty structure) or Interdisciplinary Sciences/Studies. We also provided an open option for participants to answer in their own words. The list of all the disciplines is provided in Supplementary Material 2. This survey was intended to give us a sense of the decadal change in formal training/education backgrounds of the researchers in the broader resilience research and practice network.

### Exploring the dilemma of undisciplinary science through forum theater

Forum theater has been applied in a variety of contexts to assist people in finding solutions to a wide range of challenges (Brett-MacLean et al. 2012). As its name suggests, at the core of this methodology is the development and presentation of a piece of theater, aimed at communicating a topic, idea, or situation, which is characterized by conflict or complexity. Forum theater is used to break down the traditional barrier between actors and audience members, encouraging the latter to become ‘spect-actors’ who can replace actors at points of frustration in the story, to insert their own actions and thus determine the course of the play toward a more favorable outcome (Sullivan and Lloyd 2006; Kumagai et al. 2007). We chose this method because of its ability to clearly and coherently illustrate a tangible dilemma in a neutral space, allowing for an interactive learning process to take place between the audience members and performers.

We applied the forum theater method in two steps. First, we had an internal exploratory workshop at our home institution to draw out and distill the challenges that we as PhD students face in an interdisciplinary PhD program (for details see Supplementary Material 3). The challenges identified were:

1. Breadth vs. depth in scientific knowledge—how do we ensure the broad and diverse knowledge needed for sustainability science while building and maintaining in-depth disciplinary knowledge and practice?
2. Identity as a scientist—if we do not see ourselves as ‘ecologists’ or ‘economists’ or otherwise belonging to some discipline, who are we?
3. Institutional structures—how can we pursue an interdisciplinary career within today’s university structures and professional reward systems?

Based on this first internal round of forum theater, we developed a script of a fictitious and deliberately exaggerated situation (see Supplementary Material 3) that explored a character struggling to come to terms with these challenges. This scripted play was then performed as a piece of forum theater during the session at the Resilience 2014 conference, in front of an audience of about 150 people that included a wide array of attendees, from PhD students to senior scientists. The audience participated in the theater by giving suggestions to the characters on how to respond to these challenges, which were then acted out.

Thus, the forum theater exercises helped us to evaluate not only our own experiences as PhD students (in the first, internal round), but also allowed us to clarify and illustrate the opportunities and challenges we face to a broad and diverse audience, as well as assess the reactions and interventions of the audience (in the second, public round). The three challenges identified in the internal forum theater helped us elicit questions to focus on for the panel discussion that followed.

### Panel discussion on opportunities and challenges of the undisciplinary journey

In a final step, we built on the insights gained from the survey, as well as the internal theater workshop and public forum theater session, to facilitate a deeper discussion with four invited experts in the field of sustainability science. Immediately after the forum theater session, we held a panel discussion. Our motivation for including those particular individuals is summarized in Table 2. The panel was focused on ‘the undisciplinary dilemma’ (as stated in the introduction), inter- and transdisciplinary research methodologies, and teaching and educational program design.

**Table 2:** List of panelists in the panel discussion, their relevant experience, and the questions they were asked

Panelist	Area of expertise	Questions asked
Joern Fischer, Leuphana University Lüneburg, Germany	Experience with interdisciplinary activities and research programs in the context of landscape sustainability. E.g., Fischer et al. (2012, 2014) Host of Ideas 4 Sustainability blog: https://ideas4sustainability.wordpress.com	We have heard that you are starting a new interdisciplinary research program and that you are in the process of recruiting new PhD students and Postdocs. What are the skills and competences you are looking for in that recruitment process?
Joan David Tàbara, Autonomous University of Barcelona, Spain	Experience in sustainability knowledge integration and learning, as well as reframing of research, education and policy for sustainability. E.g., Tàbara (2013a, b); Tàbara and Chabay (2013)	What is your personal survival kit for working towards knowledge integration?
Tracy Van Holt, East Carolina University, USA	Holds an interdisciplinary degree, with extensive experience in interdisciplinary scientific methodologies used in sustainability science. E.g., Van Holt et al. (2016); Brondizio and Van Holt (2014)	Regarding all the opportunities and challenges we face as interdisciplinary researchers, in your opinion, what are the kinds of competences we should develop? And how can we ‘market’ those better?
Frances Westley, University of Waterloo, Canada	Experience in interdisciplinary research collaboration as well as having led and designed novel curricula for interdisciplinary programs at the University of Waterloo. E.g., Westley et al. (2011); Westley and Antadze (2010)	Do you foresee any favorable changes in the near future regarding the institutional structure of universities, and research in general, becoming more favorable for interdisciplinary researchers to build a career?

We analyzed the data of the panel discussion based on inductively emergent themes (Boyatzis 1998), following the precepts of grounded theory (Strauss and Corbin 1998). Specifically, members of the author team identified aspects of the panel discussion transcript to answer the question: ‘What are tools or concepts that can help us best navigate the undisciplinary journey on which we find ourselves?’ The emergent themes were validated through a process of thematic consensus building between all the authors (Huberman and Miles 2002).

## Results

### The undisciplinary journey as a new phenomenon

**Fig. 1:**
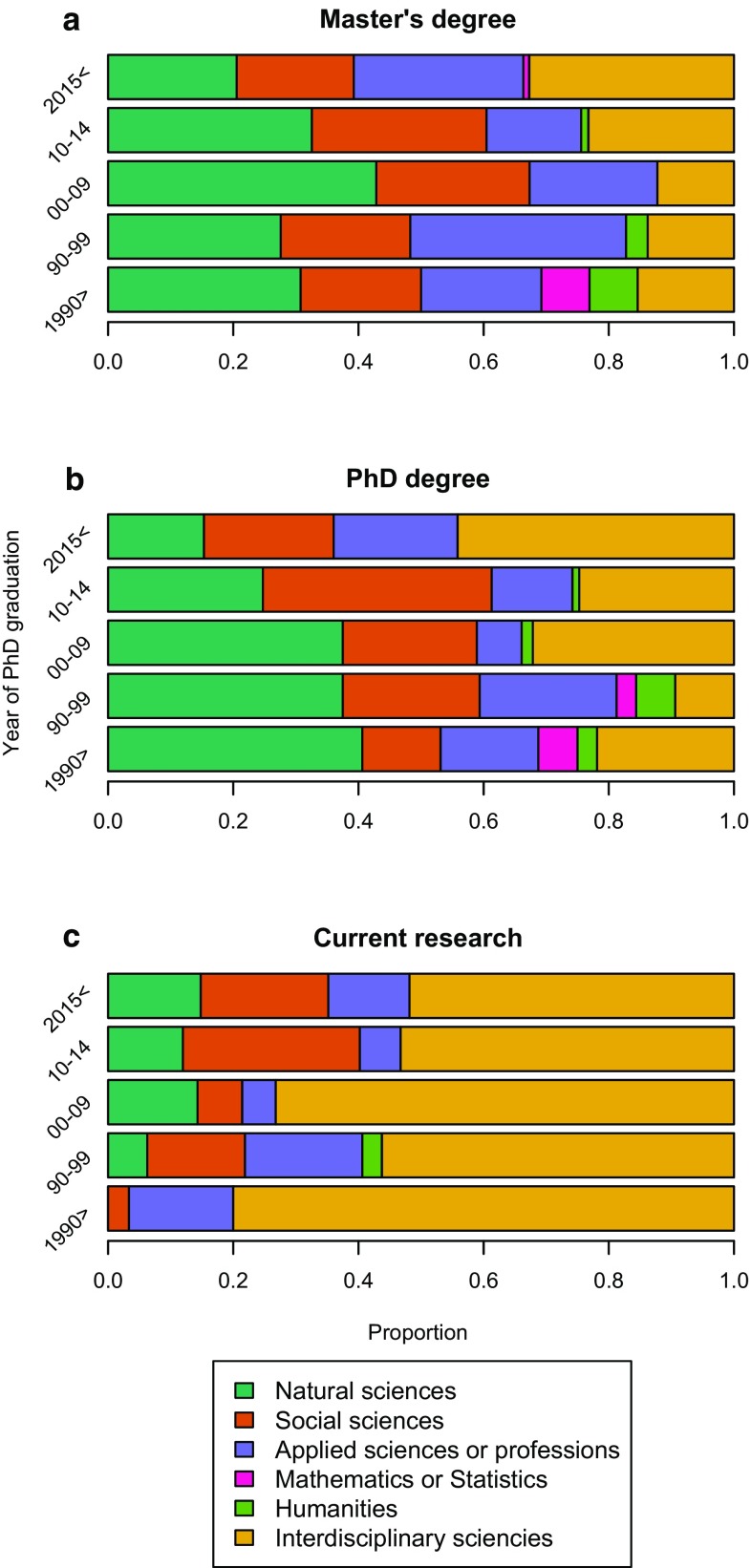
Results from survey exploring research backgrounds of Resilience 2014 conference participants. Number of respondents: before 1990: 32; 1990–1999: 32; 2000–2009: 56; 2010–2014: 93; after 2015: 111. The list of options for the online survey is found in Supplementary Material 2

The results from our exploratory survey suggest that in the current generation of sustainability science PhD students within the broader resilience research and practice network (i.e., those who had not yet obtained their degree at the time of the survey), there is a higher proportion of individuals who have an interdisciplinary academic background (at Master’s level) than in respondents who have already obtained their PhDs. Figure 1a shows how Interdisciplinary Sciences/Studies and Applied Sciences/Professions dominate over Social and Natural Science Master’s degrees for this group. Similarly, the proportion of Interdisciplinary Sciences/Studies PhD programs is the highest for PhD students who expect to obtain their degrees in 2015 or later (at the time of survey, administered in April 2014) (Fig. 1b).

Interestingly, though these PhD students described their degrees as interdisciplinary, a smaller proportion described their current research as interdisciplinary compared to older generations (Fig. 1c). One possible explanation for these results might be that today’s established sustainability scholars, who started with a more disciplinary background, are more willing to identify themselves as interdisciplinary, while an increasing fraction of the early-career researchers feel the need to describe their own work as clearly demarcated within a discipline. An alternative explanation is that senior and junior scholars perceive disciplines differently, where more junior scholars may consider sustainability science as a discipline, while senior scientists see it as interdisciplinary.

### Forum theater and panel discussion outcomes

**Fig. 2:**
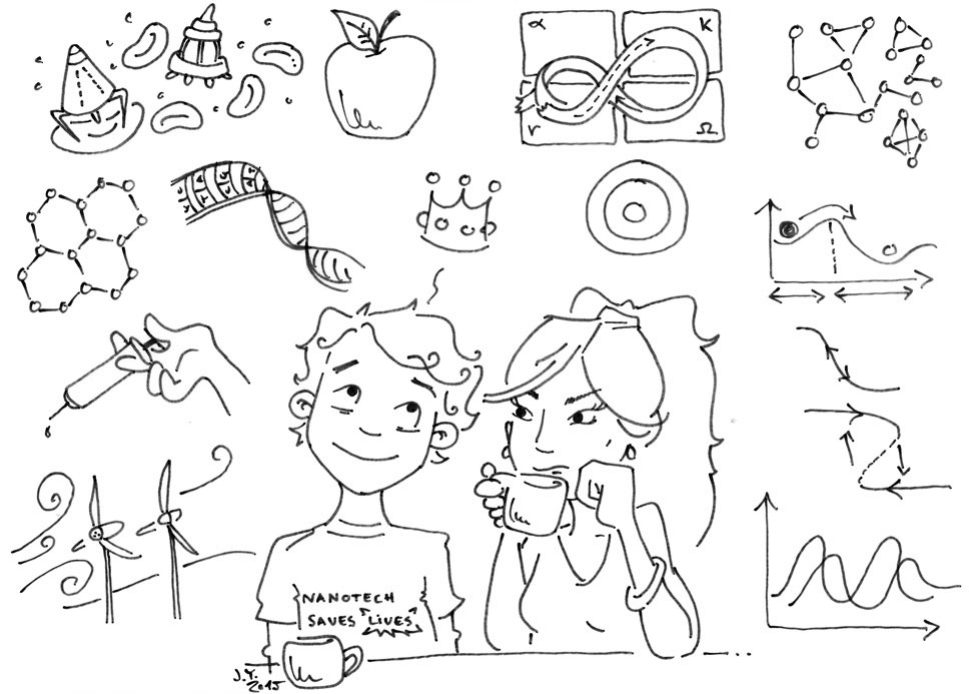
Artistic rendition of the sustainability science PhD student (*right*) attempting to explain her ‘background’ to the more disciplinary ‘Nanotech science’ PhD student (*left*) who questioned her professional identity. Audience suggestions included: ‘don’t over share, explain your research question, and stand your ground with confidence.’ The video of the forum theater performance can be seen at https://www.youtube.com/watch?v=NveKDnImxS0 (Drawing by Johanna Yletyinen)

The forum theater at the conference session prompted active audience engagement. When the protagonist struggled with her ‘*dilemma*’ (Fig. 2, and see introduction), audience members stepped in to provide suggestions on how she might navigate this uncomfortable space/situation (in which a more disciplinary scientist questioned her professional identity), how to balance breadth and depth in her research, and how to develop a niche in an interdisciplinary world (see script and link to video in Supplementary Material 3). The audience input was creative and helpful, but did not provide easy solutions.

The theater performance set the stage for the panel discussion, in which three competency streams emerged as being critical to successfully navigate the ‘undisciplinary journey’: (1) the need for a deep *grounding in methodology*; (2) the importance of being aware of and able to navigate ontological and epistemological differences (‘*epistemological agility*’); (3) the ability to strategically *navigate existing institutional spaces and structures*.

**Fig. 3:**
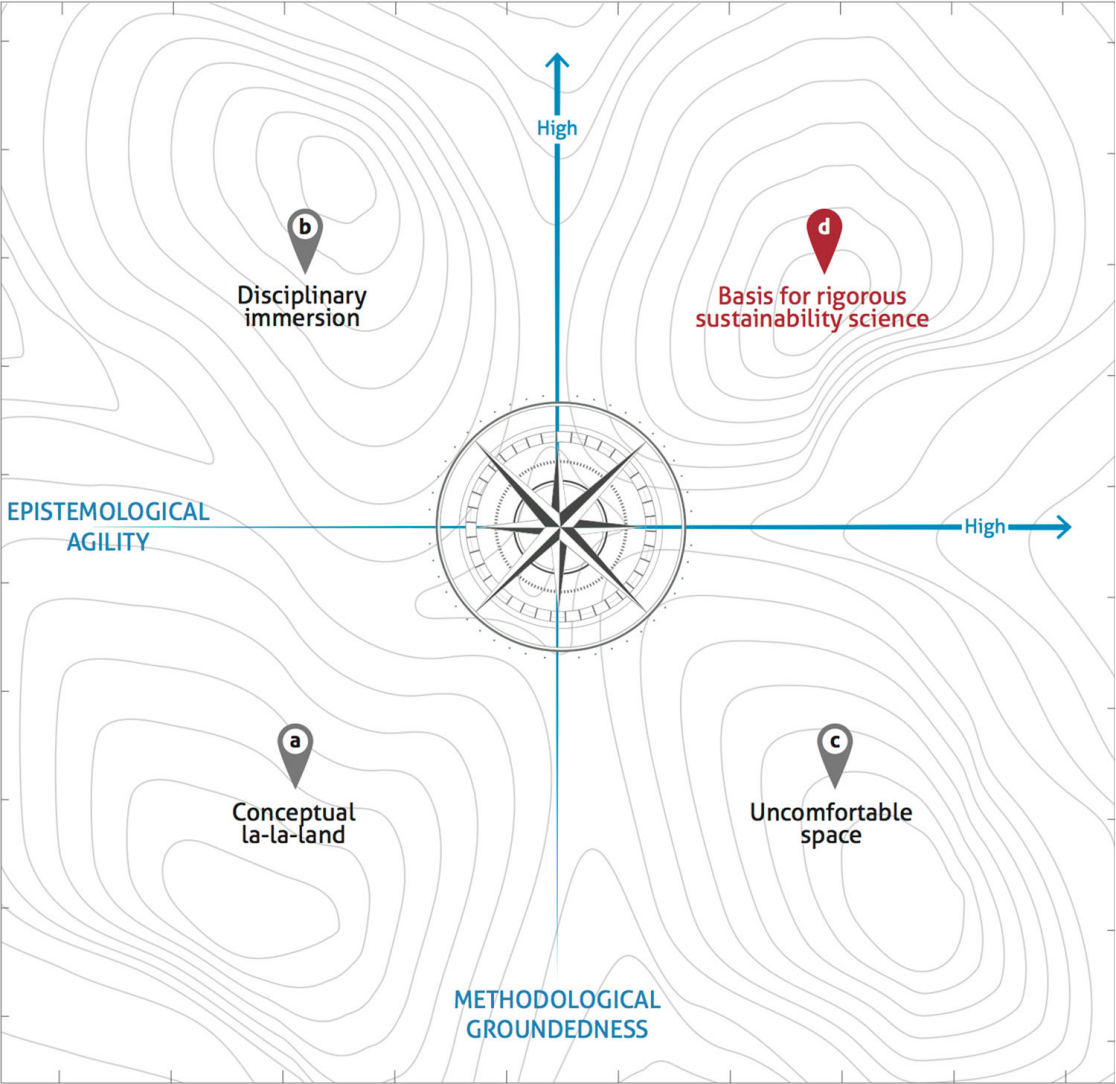
Undisciplinary compass. The relationship between two guiding competencies: epistemological agility and methodological groundedness (figure credit: Jerker Lokrantz/Azote)

Following the analysis of the panel discussion, we highlight two guiding competencies: ‘methodological groundedness’ and ‘epistemological agility’. The need to identify and develop core competencies in sustainability science is becoming increasingly recognized (Barth et al. 2007; Wiek et al. 2011). The process to develop these competencies takes place both through formal learning (through institutional support and pedagogical structure), as well as informal mechanisms (through peer engagement and students’ self-responsibility) (Barth et al. 2007). Methodological groundedness can be defined as the deep understanding and skillful handling of at least one specific methodological approach for data gathering, modeling, and/or analysis that is relevant to some area of sustainability science, but ideally is applicable to a range of areas. Based on the panelists’ input, we define epistemological agility as an understanding of different ontological and epistemological standpoints and views across multiple disciplines, enabling better communication and collaboration with different researchers, and facilitating open interdisciplinary practice for individuals and within research teams. In other words, this includes a self-reflexiveness to not only work with other disciplines, but also to work within them. This goes beyond epistemological awareness to emphasize an individual’s ability to use alternative epistemological lenses. Such agility necessarily emphasizes and places a high value on the humility and openness that should characterize engagement across epistemologies. Balancing methodological groundedness and epistemological agility can form a basis for rigorous sustainability science (Fig. 3).

These two guiding competences characterize the well-known ‘breadth’ vs. ‘depth’ struggle in science (Schwartz et al. 2008). Figure 3 illustrates how methodological groundedness and epistemological agility relate, and that there may be different ways to navigate this journey in the quest to achieve rigorous science, as an early-career scholar with interdisciplinary training.

In Fig. 3, the lower left quadrant (a) is what one of the panelists referred to as conceptual la–la-land (Table 3) where jargon and concepts can hijack early-career scholars’ attention if they have not been able to build competency in epistemological agility and methodological groundedness. The upper left quadrant (b) is what we refer to as disciplinary immersion, where methodological skills are high but reflection on the epistemological underpinning of the methods and approaches is poor, making it easy to get sucked into the strong attractor of a discipline. The lower right corner (c) is characterized by a high degree of epistemological awareness but limited skills in specific methods. We call this an uncomfortable space, where high reflexivity without a robust skill-set can cause anxiety about one’s possible scientific contribution. The upper right quadrant (d), characterized by the successful combination of high epistemological agility and methodological groundedness, is what we refer to as a basis for conducting rigorous sustainability science as an undisciplinary scholar (in red to represent compass orientation).

**Table 3:** Key quotes from panel discussion and coded themes to which they contribute

Quote	Theme
TvH: ‘Show me your method, show what you can bring. And it doesn’t matter what discipline it comes from. That is an avenue for interdisciplinary collaboration.’ JF: ‘The biggest risk I see in people that go very interdisciplinary in their PhDs is that they end up being conceptually very broad, but get stuck in what has sometimes been called “conceptual la–la-land”: They know a little bit about everything but they are not actually good at anything, and that is a real problem. That doesn’t mean that you have to be good at a particular discipline, [but rather] things that are transportable, that you can use in many different instances.’ JF: ‘My main recommendation would be not to think so much about disciplines, but think about two or three things that you are good at, and make those your profile, make those your strengths. These could be an analytical technique or writing brilliant conceptual papers, whatever it is, but you have got to have something that is unique to you. (…) have a couple of strengths that you recognize and build on those.’	Methodological grounding
FW: ‘So what is the survival kit? It is something I will call “epistemological agility” (…): basically understanding where thinking comes from. Within science and social science you have these multiple paradigms about what is truth, what is data, what is the role of the researcher. They are different from each other, but if you are trained to recognize that, it is like cracking codes. It means you know how to interface with whatever group you are looking at. (…) You start getting much more comfortable with [interdisciplinary research] once you start decoding in that way, and you come to situate yourself, and see the weaknesses in other perspectives, and your own as well. This makes you feel much more assured about interacting with multiple different types of disciplines and scientists.’ JDT: ‘Different types of problems require different types of methods. The main method in my view is conversation. Students need to be able to listen, and ask the right questions. I believe in epistemological democracy.’ JDT: ‘If you dig a hole too deep, you might not be able to get out; I think that is the problem. So try to get everything you need to know, but not more, and then move to the next thing.’	Epistemological agility
FW: ‘In this highly interdisciplinary context we ought to train our graduate students with real good courses on research design and epistemology so that they have that capacity to crack codes. (…) It is the hidden superpower, (…) if you develop some sophistication there, it gives you a leg up in discussions, which I think it is a key interdisciplinary skill.’ FW: At the moment, ‘in order to get academic jobs the vast majority will have to squeeze into a disciplinary slot. (…) There are a few institutes and think tanks within universities which search specifically for interdisciplinary training, but they are still few and far between.’ JF: ‘For PhD students that start with me, I’m not really too worried if they know much at all really, depending on what the research is they are going to have to pick new research skills as they go anyway. (…) But for a postdoc it’s a bit different. I expect a postdoc to help me, I need them to already have skills.’	Navigating institutions and education

*TvH* Tracy van Holt, *JF* Joern Fischer, *FW* Frances Westley, *JDT* Joan David Tàbara

## Discussion

### To be undisciplinary

Building on these explorations, this paper offers an early-career scholar perspective on sustainability research. In Robinson’s (2008) paper on ‘Being Undisciplined,’ different temperaments of interdisciplinarity are put forth, but what it means to be undisciplinary is never clearly defined. Based on our experiences and the results presented here, we provide a working definition of undisciplinary to describe the journey that early-career researchers navigate in order to achieve rigorous sustainability science. Undisciplinary science is therefore not a new type (or further evolution) of mixed-disciplinary research [i.e., it does not replace multi-, inter-, or trans-disciplinarity (MIT-disciplinarity) see Klein (2017)], rather it is a personal process as well as a mode of collaboration from which one can engage in various strategies of research. The types of research and the particular contexts that we face as early-career researchers create an undisciplinary space that is part of our unique MIT-disciplinary process, and the reflexive undisciplinary process and orientation provides us with an identity and a set of skills to engage in MIT-disciplinary research. We use this lens to unpack our core dilemma and what it means to be early-career researchers, with interdisciplinary training while engaging with others with similar backgrounds, in a world dominated by established disciplines.

### Undisciplinary journey

In attempting to define undisciplinary, we asked ourselves these questions: Is an undisciplinary space something that early-career sustainability researchers find themselves in and have to deal with? Or is it something that we would like to embrace as a quality that improves our research and its impact? We find that it can be simultaneously understood as a space and a process, which together form the undisciplinary journey. In dealing with what it means to navigate an undisciplinary space, undisciplinary scholars go through a process, which is our primary focus in this paper. We build on four distinct phases or professional situations in which early-career scholars may find themselves, and describe three main stylized trajectories between them (Fig. 3, quadrants a–d), which we argue can help to guide an undisciplinary journey within sustainability science. Finally, we discuss the undisciplinary journey as something that can be developed as an asset by individuals or groups of sustainability scholars. In viewing and defining this journey as a space, a process and an orientation, we hope to convey that an undisciplinary orientation can ultimately become an asset that enables rigorous, solutions-oriented science within groups of scholars and institutions. The section concludes with suggestions for institutional support for undisciplinary research journeys and endeavors.

### Undisciplinary space

The dilemma articulated at the beginning of this paper was: How could we ‘perform high-quality research and build identity in the field of sustainability science, when starting as interdisciplinary individuals without profound roots in a discipline, and working within a world dominated by established disciplines?’ This undisciplinary space, created through the research questions we pose, is a space in which there are no clear rules or forms of engagement, no writing formulas, nor clear methodological pathways. Sword (Sword 2012, p. 12), in her book aimed at graduate students, on *Stylish Writing*, states that, conventionally, ‘to enter an academic discipline is to *become* disciplined: trained to habits of order through correction and chastisements that are ‘assumed to be salutary’ by one’s teachers’. The undisciplinary space is the arena in which the larger undisciplinary journey happens (Fig. 3), a space in which there are no boundaries to guide you, but also no boundaries to hold you back.

### Undisciplinary process

Central to this journey is the undisciplinary process. This process involves navigating through the undisciplinary space depicted in Fig. 3. We propose that a necessary characteristic to follow a fruitful undisciplinary process of scholarship is self-reflexivity, which involves examining both changes in oneself and the relationship between research process and outcomes (Hsiung 2010). The undisciplinary compass (Fig. 3) provides guidance for constantly re-evaluating where you are in the research process, and what skills you may need to seek in collaborators, or hone for yourself.

Viewing the different quadrants as states for individual researchers (or research groups) suggests that there are different ways to transition from one space to another, toward the goal of rigorous sustainability science (or even interdisciplinary science more broadly), which we argue can only be achieved once the individual researcher (or research group) finds themselves in the upper right quadrant. We emphasize that we see the progression and navigation between quadrants throughout a scientific career as iterative. Progressing along the horizontal axes of Fig. 3 follows a more traditional path toward rigorous interdisciplinary sustainability science. Starting from disciplinary immersion (b): Researchers are trained in a discipline, and once established in it, they decide to engage with researchers from other disciplines. Alternatively, progressing along the vertical axes of Fig. 3 represents a growing proportion of the cases in sustainability science, relative to the former trajectory, and hence we elaborate below based on insights from the panel discussion.

To illustrate the way in which we have experienced this process ourselves, imagine an early-career sustainability scholar starting her/his career with low epistemological agility and low methodological groundedness (Fig. 3a): ‘The biggest risk I see in people that go very interdisciplinary in their PhDs is that they end up being conceptually very broad, but get stuck in what has sometimes been called “conceptual la–la-land”, they know a little bit about everything but they are not actually good at anything, and that is a real problem’ (JF, quote, Table 3). Alternatively, an early-career sustainability scholar might be trained in a few core methods, but get sucked into the strong attractor of a discipline, making it hard to engage in deep interdisciplinary production (Fig. 3b). One suggested strategy to avoid the attractor of a discipline was reflected by a respondent during the panel discussion: ‘My main recommendation would be not to think so much about disciplines, but think about two or three things that you are good at, and make those your profile, make those your strengths. These could be an analytical technique or writing brilliant conceptual papers, whatever it is, but you have got to have something that is unique to you. (…) have a couple of strengths that you recognize and build on those (JF, quote, Table 3).’

A third situation would be if the early-career scholar is confident in navigating different epistemologies, but may find herself/himself in an uncomfortable space (Fig. 3c) of negotiating between different ways of producing scientific knowledge without having the methodological skills and confidence to move forward in any particular direction (especially when a direction is largely unexplored, which is quite characteristic of a new scientific field). Although we would expect that all scholars feel uncomfortable at different times in their careers, we think that the lack of disciplinary anchoring makes this feeling more poignant. Scientific progress depends on the continuous creation of uncomfortable spaces (Rayner 2012) and innovation exists at the boundary of this discomfort while at the same time finding the confidence to move forward in a robust and rigorous way.

We acknowledge and value the pioneering work done by previous generations in creating an interdisciplinary environment for sustainability research. It is in this environment that interdisciplinary graduate programs have been established around the world, in response to the demands of the evolving field of sustainability science. Our survey results suggest that now, more than ever before, PhD students are starting their careers with an interdisciplinary background. We argue that this is an important difference between the path taken by previous generations of sustainability scientists, who typically started their careers by practicing disciplinary science, then moving on to multi-, then inter- or even transdisciplinary science. Achieving undisciplinary orientation is by no means a homogenous identity. Some researchers may find themselves with strong epistemological agility and may have opted for methodological pluralism (Norgaard 1989) as opposed to others with more methodological depth.

Navigating this balance between depth and breadth demands an array of more specific skills, which will vary across fields of inquiry. An early-career scholar, sustainability science group, or program, makes trade-offs at different points in time between learning in-depth methods, reading broadly on different ways of knowing, and developing other competencies to do rigorous interdisciplinary science. Core competencies in sustainability science have been proposed by Wiek et al. (2011: systems thinking, anticipatory, normative, strategic and interpersonal competencies). In addition to these competencies we find it helpful to think of specific skills along the continuum of our broader guiding competencies of epistemological agility and methodological groundedness. On the one hand, they are specific technical skills, such as geographical information systems (GIS) analysis, network analysis, statistical or mathematical modeling, qualitative data analysis, interviewing skills. Other skills include collaborative interpersonal skills across cultures, between ideologies and working in different contexts. Skills in facilitation, participatory approaches and synthesis (i.e., to see and make sense of ‘the big picture’) are also valuable skills in sustainability science. Some skills and methods align better with certain epistemologies (West et al. 2014). High epistemological agility would allow a researcher to effectively select appropriate and relevant methodologies according to the specific research needs. In line with our proposed undisciplinary process, epistemological agility means the ability to discern different disciplinary traditions and navigate between them with confidence in order to match ontologies with appropriate epistemologies and methodologies (Table 3; McWilliam 2012; Khagram et al. 2010). Agility should help scholars avoid getting stuck in certain theoretical approaches or scientific paradigms, but equally important is the awareness and openness to different ways of knowing and learning, which is critical for addressing issues of social-ecological complexity (personal communication Joan David Tàbara). Through this undisciplinary journey, early-career scholars may come to acquire a foundation for doing rigorous sustainability science, balancing methodological groundedness and epistemological agility.

### Undisciplinary orientation

Navigating the undisciplinary process, we can develop an undisciplinary orientation: the ability and desire to embrace the undisciplinary journey. It goes beyond accepting discomfort at the boundaries of disciplines, and science, more broadly. An undisciplinary orientation is to embrace complexity and uncertainty in the pursuit of problem-oriented research.

In addition to the core competencies and specific skills of engaging in rigorous sustainability science, the successful navigation of the process is a quality of its own right, which should be nurtured and acknowledged within the growing institutional structures around sustainability science, at various different phases of a researcher’s career. Embracing this orientation may be particularly helpful for early-career scholars dealing with an undisciplinary space, and processing their own dilemmas. Indeed, as a group of authors and cohort of PhD students, our reflexive engagement with this journey has contributed to clarifying our individual research identities and contributions to sustainability science.

### Methodological reflections

The tension and obstacles inherent in combining very different methods, potentially even based on different epistemologies, characterizes the journey toward building a foundation for rigorous sustainability science. Arts and performative methods have played a key role in challenging existing epistemologies and identities (Heras and Tàbara 2014) and provide an arena to ‘open up’ knowledge systems (Cornell et al. 2013). The process of combining diverse methods, including performative methods, in this paper was in itself a valuable and constructive learning experience and is representative of the types of challenges faced by early-career interdisciplinary scholars in their collaborations with colleagues from either other disciplines and/or others with similarly interdisciplinary backgrounds. Indeed, the challenge of such a methodological approach was evident in discussions between the authors in developing and presenting the methodologies used and the results in this paper.

The forum theater, the thematic analysis of a panel discussion from a conference session, and the survey complement each other and are indicative of the methodological pluralism and innovative combination of methods that is characteristic of interdisciplinary sustainability science (Norgaard 1989; Folke et al. 2016). Each method served a specific purpose and built on each other in the following way:

1. The survey provided support that there has been a ‘generational shift’ with respect to the backgrounds and starting points of those pursuing interdisciplinary sustainability science.
2. The forum theater provided a structure that encouraged openness, and reflexivity for, and insights into, interdisciplinary scholarship from early-career scholars, while also incorporating experiences and reflections from the (more experienced) audience.
3. The panel built on these experiential insights through expert elicitation.

The opportunity for engagement by the authors over a period of three years in articulating a joint dilemma, designing the methodologies to explore it, and lengthy discussions throughout the writing process of this paper, created an arena for personal and collective reflexivity through which we came to understand and think strategically about the undisciplinary journey. The phenomenon of an undisciplinary journey, as first and foremost a process, but also a space and an orientation, which we describe in this paper in relation to sustainability science, is not a new ‘discovery’ and is indeed part of the cycle of science (Kuhn 1962), where real-life challenges arise that single disciplines are not able to address and thus new disciplines or collaborative fields are born. Yet, as part of this cycle, Bettencourt and Kaur (2011) and Kates (2011) contend that sustainability science is a new, and different kind of science. In viewing sustainability science as a dynamic, post-normal science that should not be reduced to traditional disciplinary boundaries, our reflection on the undisciplinary journey can help make sense of this space through engaging in self-reflexive processes and collaborations. As more and more interdisciplinary scholars enter graduate programs with interdisciplinary backgrounds, institutional structures may choose to reflect on and embrace undisciplinary orientations to help reduce the inherent risks and challenges involved in pursuing highly interdisciplinary PhDs.

### Academic institutions—navigation aids for undisciplinary processes

Becoming an accomplished sustainability researcher, or even more broadly an interdisciplinary scholar, involves an inevitable process of iteration, through combining and matching different methodologies with different epistemologies in order to best address any given problem. There is an important role for institutions to play in this process, both in training and educating early-career scholars in order to minimize the time spent in an ‘uncomfortable space’ or in ‘conceptual la–la-land,’ as well as creating more promising and substantive career trajectories.

Interdisciplinary research centers and departments are increasingly offering interdisciplinary PhD programs (see Supplementary Material 1), and it is critically important to ask how these programs can pedagogically support PhD students in their navigation of an undisciplinary journey. What are the opportunities and challenges, for example, in being supervised by researchers who themselves were trained in disciplinary schools? What are the risks involved in being examined by scholars established in disciplinary traditions? Ideas about what an interdisciplinary skill-set in sustainability science looks like remain nascent as the field itself is still emerging. Inevitably there will be different perspectives on how best to do this; the panelists providing expert advice in this paper also had somewhat contradictory ideas on the pedagogical strategy of educating PhD students, with one advocating learning-by-doing, and another emphasizing the importance of formal training in epistemological agility (Table 3).

In either case, a pedagogical strategy should align with an awareness of the outlets that exist for publication as well as future career opportunities. There is an increasing recognition of the continuing development of high-quality interdisciplinary research in the publishing sphere, the creation of the sustainability science section in *PNAS* (Clark 2007), the creation of the journal *Sustainability Science* (Komiyama and Takeuchi 2006), the soon to be launched *Nature Sustainability* journal, and the early recognition of sustainability science as a science of its own (Kates et al. 2001). Despite these positive steps toward publications in sustainability science, a recent paper in *Nature* suggests there is a growing gap between the increasing number of possibilities to conduct interdisciplinary research and the level of career advancement, since most academic institutions still place more value on high profile research outputs like articles in high-impact journals than other outcomes like cooperative learning or policy engagement (Gewin 2014).

## Conclusions

Our results from the survey, forum theater, and panel discussion indicate that a growing proportion of the sustainability science research community can be described as ‘undisciplinary’ scholars, based on their interdisciplinary training, and who consequently face new opportunities and challenges as early-career sustainability researchers. In the paper we share our experiences in facing an undisciplinary journey and suggest guides to navigate and embrace this process. We propose the undisciplinary journey as a new and as yet relatively uncharted journey for achieving rigorous inter- or transdisciplinary sustainability research and propose an ‘undisciplinary compass’ to help navigate this journey toward more sustainable futures through reflexively balancing methodological groundedness with epistemological agility. The undisciplinary journey and compass we describe may also be helpful for other interdisciplinary fields, or for scholars who may have abandoned their ‘background’ discipline and find themselves in new and uncomfortable spaces. We hope that the exploratory insights offered in this paper provide useful suggestions to early-career researchers, research teams and interdisciplinary research centers to facilitate navigation of the ever-evolving boundaries within sustainability science, where innovations and solutions emerge.

## Electronic supplementary material

Below is the link to the electronic supplementary material.

Supplementary material 1 (DOCX 213 kb)

Supplementary material 2 (DOCX 77 kb)

Supplementary material 3 (DOCX 138 kb)
