# Supplementary material for: The undisciplinary journey: early-career perspectives in sustainability science
Source: Sustain Sci. 2017 Jun 21;13(1):191–204. doi: 10.1007/s11625-017-0445-1 (PMC6086269; doi:10.1007/s11625-017-0445-1)
Supplement: Supplementary file 1 — Supplementary material 1 (DOCX 213 kb) [file 11625_2017_445_MOESM1_ESM.docx]

**Supplementary Material 1 (S1)**

**Non-exhaustive list of SES graduate programs (from EANTH list-serv)**

1. Rutgers University Dept. of Ecology's Human Dimensions of Environmental Change Graduate Certificate Program <http://www.humanecology.rutgers.edu/gradStudyCert.asp>
2. University of Manitoba's Natural Resources Institute [http://umanitoba.ca/institutes/natural_resources/http://umanitoba.ca/institutes/natural_resources/](http://umanitoba.ca/institutes/natural_resources/)
3. University of Maine's School of Marine Sciences <http://www.umaine.edu/marine/programs/graduate-programs.php>; Also their Anthropology and Environmental Policy degree [http://umaine.edu/anthropology/degree-programs/phd-in-anthropology-environmental-policy/](http:///h)
4. University of Georgia's Integrative Conservation (ICON) Graduate Program <http://icon.uga.edu/>
5. Yale School of Forestry and Environmental Studies' combined Forestry and Environmental Science (F&ES)/Anthropology Ph.D. Program <http://environment.yale.edu/doctoral/degrees/combined-anthropology/>
6. Indiana University's Anthropological Center for Training and Research on Global Environmental Change <http://www.indiana.edu/~act/>
7. Michigan State University's Center for Systems Integration and Sustainability [http://csis.msu.edu/http://csis.msu.edu/](http://csis.msu.edu/)
8. Stanford University's Emmett Interdisciplinary Program in the Environment and Resources <https://earth.stanford.edu/eiper> and Stanford's Ecology and Environment track within the Dept of Anthropology <https://web.stanford.edu/dept/anthropology/EEsites/>
9. Duke University's Nicholas School of the Environment's Marine Lab <http://nicholas.duke.edu/marinelab>
10. Stockholm Resilience Centre <http://www.stockholmresilience.org/education/resilience-research-school.html>
11. U of New Hampshire's Dept. of Natural Resources and Environment. Several M.S. degrees and a Ph.D. in Natural Resources and Earth Systems Science <http://www.nre.unh.edu/graduate-programs>
12. University of Idaho's Resilience Thematic Group includes an interdisciplinary Water Resources graduate program <http://www.uidaho.edu/cogs/envs-wr/academics/water-resources> (in particular the IGERT focused on adaptationhttp://[www.uidaho.edu/cogs/envs-wr/academics/water-resources/igert-program](http://www.uidaho.edu/cogs/envs-wr/academics/water-resources/igert-program)) and a current EPSCoR grant, MILES (Managing Idaho’s Landscapes for Ecosystem Services) is also focused within a social-ecological framework (<http://vivo.nkn.uidaho.edu/vivo/display/n20059>)
13. University of Florida's Masters of Sustainable Development Practice
14. <http://mdp.africa.ufl.edu/about/>
15. University of Aberdeen's MSc in People and the Environment <http://www.abdn.ac.uk/study/courses/postgraduate/taught/people_environment/>
16. Ohio State's Dept. of Anthropology. Scroll down to overview of graduate degrees in Cultural Anthropology: [https://anthropology.osu.edu/grad/fields#Chttps://anthropology.osu.edu/grad/fields - C](https://anthropology.osu.edu/grad/fields#C)
17. Prescott College's PhD in Sustainability Education: <https://www.prescott.edu/academics/phd-sustainability-education/>
18. U of Washington's School of Marine and Environmental Affairs -- with Marc Miller <http://depts.washington.edu/smea/content/areas-concentration>; also Environmental Management Certificate [http://poe.washington.edu/graduate_programs/environmental-management-certificate/index.phphttp://poe.washington.edu/graduate_programs/environmental-management-certificate/index.php](http://poe.washington.edu/graduate_programs/environmental-management-certificate/index.php)
19. U of Wisconsin's MS in Natural Resources: Environmental Education & Interpretation <http://www.uwsp.edu/cnr/AMP/pages/default.aspx>
20. University of Wisconsin-Madison Community/Environmental Sociology program  [http://dces.wisc.edu/programs/graduate-program/](http:///h)
21. Arizona State University - programs in Sustainability (<https://schoolofsustainability.asu.edu/graduate/graduate-degree-programs.php>) and Environment Social Sciences (<https://shesc.asu.edu/graduate/phd-program/environmental-social-science>). Available to students in both programs (and several others) is a complex adaptive systems concentration (<https://shesc.asu.edu/graduate/complex-adaptive-systems-science-concentration>)
22. U of British Colombia's Institute for Resources, Environment and Sustainability -- with Terre Satterfield <http://ires.ubc.ca/>
23. The Pontificia Javeriana's School of Rural and Environmental Studies:<http://puj-portal.javeriana.edu.co/portal/page/portal/Facultad%20de%20Estudios%20Ambientales%20y%20Rurales/INICIO/home_estudios_amb>
24. U. Colorado, Boulder has a degree in Anthropology that can include a focus on landscape and space (<http://anthropology.colorado.edu/about-us/full-vision-statement/>); also the Institute for Behavioral Sciences has a Center for the Governance of Natural Resources that includes an Environment and Society Program (<http://www.colorado.edu/ibs/cgnr/>)
25. University of South Florida, offers a Phd in Applied Anthropology (<http://www.grad.usf.edu/programs/programinfomation.php?pcode=APAASBCMPh.D>.) and a certificate in environmental planning (<http://www.usf.edu/innovative-education/programs/graduate-certificates/environmental-policy-and-management.aspx>)
