# Supplementary material for: The undisciplinary journey: early-career perspectives in sustainability science
Source: Sustain Sci. 2017 Jun 21;13(1):191–204. doi: 10.1007/s11625-017-0445-1 (PMC6086269; doi:10.1007/s11625-017-0445-1)
Supplement: Supplementary file 2 — Supplementary material 2 (DOCX 77 kb) [file 11625_2017_445_MOESM2_ESM.docx]

**Supplementary Material 2 (S2)**

Table S2 List of options for the online survey.

| **Discipline** | **Category** |
| --- | --- |
| Anthropology or Archaeology | Social sciences |
| Economics | Social sciences |
| Geography | Social sciences |
| Gender studies | Social sciences |
| Political science | Social sciences |
| Psychology | Social sciences |
| Sociology | Social sciences |
| Chemistry | Natural sciences |
| Earth sciences | Natural sciences |
| Environmental sciences (including Ecology) | Natural sciences |
| Life sciences | Natural sciences |
| Physics | Natural sciences |
| Space sciences | Natural sciences |
| History | Humanities |
| Linguistics | Humanities |
| Literature | Humanities |
| Performative or Visual Arts | Humanities |
| Philosophy | Humanities |
| Agricultural or Forestry sciences | Applied Sciences or Professions |
| Architecture or Design | Applied Sciences or Professions |
| Business management | Applied Sciences or Professions |
| Environmental studies | Applied Sciences or Professions |
| Engineering or Computer sciences | Applied Sciences or Professions |
| Law or Public administration | Applied Sciences or Professions |
| Medicine or Healthcare science | Applied Sciences or Professions |
| Mathematics or Statistics | Mathematics or Statistics |
| Interdisciplinary sciences/studies, including multiple of the above listed or others | Interdisciplinary sciences/studies |
| Two or more degrees | Interdisciplinary sciences/studies |
