# Supplementary material for: The undisciplinary journey: early-career perspectives in sustainability science
Source: Sustain Sci. 2017 Jun 21;13(1):191–204. doi: 10.1007/s11625-017-0445-1 (PMC6086269; doi:10.1007/s11625-017-0445-1)
Supplement: Supplementary file 3 — Supplementary material 3 (DOCX 138 kb) [file 11625_2017_445_MOESM3_ESM.docx]

**Supplementary Material 3 (S3)**

**Detailed description of the forum theater workshop at the Resilience 2014 conference.**

We started the session at the Montpellier conference by presenting the survey results, i.e. the same as in Fig. 1 in this paper. After that, the play begun, based on the below script. The play was about 10 minutes in total. The audience were invited to break at any time and come with suggestion to the student on how to respond to the newcomer.

After a few iterations, we finished the theater session and immediately invited the panel.

**Script of forum theatre play at the Resilience 2014 conference**

Stories from the front – reportage on the life of a trans-disciplinary PhD Student (10-15 Minutes)

The Players: A Narrator (CS), A trans-disciplinary PhD student (LJH), The Newcomer; A biomedical PhD student (AM) and an inner voice (MH).

*It is a beautiful morning and our keen PhD student sets out confident that she is contributing to making the world a better place. She has been invited to speak at a large scientific conference and her nervousness and excitement are having an epic battle in her brain. She arrives, idling and looking around, a nervous smile on her lips but not in her eyes… A fellow conference attendee moves towards her.*

Newcomer: Hi there, how are you? Excited about the conference? What are you doing here, what is your research background?

PhD Student: Yes I am excited! This is my first conference; I am presenting my work here. As for my background, Well… I….um….I did my undergrad in marine biology, then I did a masters in international environmental law and now I am doing my PhD on cultural ecosystem services and development in West Africa.

Newcomer: right so you are a marine biologist then, are you looking at the culture of fish and seaweed in West African ecosystems?

PhD Student: No, well… that was my bachelors; I have moved on from there, I am not doing so much with Marine Biology now. [*An uncomfortable silence as our PhD breaks eye contact and casts her eyes towards the floor, looking up after a time she says…*] So…um, what is your background?

Newcomer: I am a molecular biologist and I am trying to cure cancer with nanotechnology tailored to the genetic makeup of individuals.

PhD Student: Oh wow, that is impressive, I hope to contribute too in my own way…[*said in a fast and frantic pace, almost stumbling over the words*] you know, cause ecosystem services are really important in enhancing adaptive capacity, especially in the renewal phase of the adaptive cycle and can contribute to poverty alleviation, breaking social ecological traps and helping communities towards transformation, and you know innovation and institutional entrepreneurs…

Newcomer: Right I did not really understand what you just said; I understood the words but when you put it together, it does not really make sense. That is what I like about my research, it is very clear how I am making the world a better place, it must be hard to not really be doing real science, how exactly do you foresee that the work you are doing will change the world?

PhD Student: Well, um, yeah, I don’t really know but like ecosystems are like our life support and local communities should be like stewards, but it is hard because of poverty and degradation of ecosystem services, [*speeds up, a trapped look in her eye*], and so understanding all that is important for the world in the future, yeah…

Newcomer:…Anyway, I want to go talk to Professor Stark who has just arrived, he was the person who undertook the foundational research for the use of nanotechnology in cancer treatment and I really don’t want to miss him, so I am going to go. Good luck with those ecosystem services and maybe see you later!

*After this infuriating conversation, our PhD student spent some time in a corner drinking bitter coffee agonising over how she needed to make her research more globally applicable. As she struggled, her idol, the superstar Professor Frau Doctor Buzzy Folkstrom approached, said she had read one of the PhD student’s papers and offered her to be on a forthcoming paper as a co-author! She stood in stunned silence, tilting her head to the side like a curious puppy.*

*After a while, when she could move again, A conversation began inside hear head...*

PhD Student: [*introspectively*] this conference is making me realise that my research needs to have much more impact on ‘real’ problems… I have been offered the chance to be part of a paper that is about the global importance of answering these critical issues.

Inner voice: [*hesitantly*]. That is important but are you really sure that should be a priority just now? I mean your last paper was rejected, twice, because your grasp of multivariate statistics was not up to the task, I think you should be spending more time deepening your knowledge of methods and not looking at the big picture all the time.

PhD Student: But I, um, I think it could be a really good opportunity and well I mean, I love statistics but, you know you said that it is important to….

Inner Voice: But you know by the time you finish your PhD you need to have developed an area of specialised knowledge. After your PhD, then you can focus on these papers that focus on the big picture.

PhD Student: [*her temper flares and she says the following defensively and with heat in her voice*] But! we are constantly told to be ‘trans-disciplinary’ How am I supposed to balance doing good robust research and still being globally ground breaking? I mean it seems like such a difficult balance...

*Our PhD student is frustrated and unsure of herself, she wishes she could go back in time and change how she dealt with the first situation and change her inner dialogue. But wait she can! Audience we call on you, how can we together work towards addressing the dilemmas of being a trans-disciplinary student of sustainability science in 2014?*

**See the video for the whole session here:** [https://www.youtube.com/watch?v=NveKDnImxS0https://www.youtube.com/watch?v=NveKDnImxS0](https://www.youtube.com/watch?v=NveKDnImxS0)

<https://www.youtube.com/watch?v=NveKDnImxS0>

<https://www.youtube.com/watch?v=NveKDnImxS0>

<https://www.youtube.com/watch?v=NveKDnImxS0>

<https://www.youtube.com/watch?v=NveKDnImxS0>
